# Horse owners’ knowledge, and opinions on recognising colic in the horse

**DOI:** 10.1111/evj.13173

**Published:** 2019-09-23

**Authors:** A. Bowden, J. H. Burford, M. L. Brennan, G. C. W. England, S. L. Freeman

**Affiliations:** ^1^ School of Veterinary Medicine and Science University of Nottingham Nottingham UK

**Keywords:** horse, owner, colic, signs, decision‐making, knowledge

## Abstract

**Background:**

Colic is the most common emergency problem in the horse. An owner’s ability to recognise colic and seek assistance is a critical first step in determining case outcome.

**Objectives:**

The aim of this study was to assess horse owners’ knowledge and opinions on recognising colic.

**Study design:**

Cross‐sectional study.

**Methods:**

An online questionnaire was distributed to horse owners with open and closed questions on their knowledge of normal clinical parameters in the horse, confidence and approach to recognising colic (including assessment through case scenarios), and their demographics. Descriptive and chi squared statistical analyses were performed.

**Results:**

There were 1564 participants. Many respondents either did not know or provided incorrect estimates for their horse’s normal clinical parameters: only 45% (n = 693/1540) gave correct normal values for heart rate, 45% (n = 694/1541) for respiratory rate and 67% (n = 1028/1534) for rectal temperature. Knowledge of normal values was statistically associated with participants’ educational qualifications (P<0.01). Owners said if they suspected their horse had colic they would assess faecal output (76%; n = 1131/1486), gastrointestinal sounds (75%; n = 1113/1486), respiratory rate (65%; n = 967/1486) and heart rate (54%; n = 797/1486). There was a lack of consensus on whether to call a vet for behavioural signs of colic, unless the signs were severe or persistent. The majority of participants (61%) were confident that they could recognise most types of colic. In the case scenarios, 49% were confident deciding that a surgical case had colic, but 9% were confident deciding an impaction case had colic.

**Main limitations:**

Most respondents were UK based; risk of self‐selection bias for owners with previous experience of colic.

**Conclusions:**

There was marked variation in horse owners’ recognition and responses to colic, and significant gaps in knowledge. This highlights the need for the development of accessible educational resources to support owners’ decision‐making.

## Introduction

Colic is the most common reason for emergency veterinary call outs to horses 1. Horse owners are essential in the recognition of colic, as they often have primary responsibility for identifying signs and deciding to seek veterinary intervention. Common reasons for a delayed response to a clinical problem are a lack of understanding or knowledge 2, 3. Investigation of horse owners’ baseline knowledge of colic, their motivations and obstacles for seeking veterinary assistance and their responses to different clinical signs of colic is essential to identify gaps in current knowledge and barriers to decision‐making.

The aim of this study was to explore horse owners’ knowledge, understanding and experience with equine colic, and describe factors that affect horse owners’ approach to a horse with clinical signs of abdominal pain. The objectives of the study were: 1) To assess horse owners’ knowledge of normal parameters in horses, and evaluate how they respond to changes in clinical and behavioural parameters in their horse; 2) To assess horse owners’ knowledge and understanding of colic, and evaluate how they would respond to different signs of colic; 3) To determine how horse owners accessed information and resources, and their experience with equine colic.

## Materials and methods

### Sample population

A cross‐sectional study was conducted with the target population being all horse owners. The sampling frame encompassed horse owners (with no restriction on length of horse ownership or level of experience), horse carers (e.g. who had a horse on loan) or those who had previously owned a horse. The questionnaire was distributed online, with convenience sampling through equine social media sites based in the UK and US, and a hard copy version was available upon request.

### Development of the questionnaire

The questionnaire was piloted with three horse owners and seven veterinary surgeons. The framework of the questionnaire was six sections: Information and consent; A. Owners’ understanding and recognition of the ‘normal’ horse; B. Owners’ understanding and recognition of colic in the horse; C. Personal experiences with colic; D. Owner rating of their confidence in recognising colic using different case scenarios; E. Owners’ demographic information and their opinion of their working relationship with their veterinarians.

The questionnaire consisted of open and closed questions, using different formats (Supplementary Item 1). A short introductory paragraph was included at the start of each new section, highlighting the need for participants to answer the questions honestly, without using resources to assist them. When asked about critical cases, this was defined as a horse that had required referral treatment for a medical or surgical condition, or been euthanased or died. Participants’ identification of colic in the horse was evaluated by the use of three different colic case scenarios (a case showing severe signs consistent with a surgical/strangulating lesion, a mild medical idiopathic condition and a pelvic flexure impaction case scenario). They were asked to select from five options (‘It definitely has colic’; ‘I think it has colic’; ‘I’m not sure if this is colic or not’; ‘I don’t think it has colic’; ‘It definitely hasn’t got colic’). Free text boxes were provided at the end of each section for any further comments, and there was an additional free text feedback section at the end of the questionnaire. The questionnaire was distributed through an online survey tool (SurveyMonkey)^1^.

### Dissemination of the survey

The survey was disseminated through the research group’s social media accounts on Facebook and Twitter, sent to 196 UK veterinary practices that had previously been involved with the research group and consented to future contact, and distributed to equine and veterinary organisations and media outlets in the UK and US. The time frame for data collection for the study was 16 weeks, from May 2014 until September 2014.

### Data analysis

Quantitative data were subject to descriptive analysis for preliminary exploration of the data, including mean, median and range for continuous variables, and percentage frequencies and mode for categorical variables. Free text responses were reviewed and categorised into themes. Continuous data were grouped into biologically meaningful categories prior to chi‐squared analysis for association with knowledge of normal clinical parameters and decision‐making outcomes. Evidence of association was accepted at P<0.05. Complex statistical analysis was not performed due to limited robustness of data collected by survey analysis.

The reference ranges used to define normal heart rate, respiratory rate and rectal temperature in this study were generated by reviewing ranges described across five reference textbooks and utilising the maximum ranges from these 4, 5, 6, 7, 8. Descriptive analysis of data was performed using Microsoft Excel and statistical analysis was conducted using SPSS V22.0^2^.

## Results

### Survey distribution and responses

There were 1564 horse owners who participated in the survey, with 1331 completing the survey in full (85% completion rate). The primary social media posts (Facebook and Twitter) were shared by BEVA, RCVS Knowledge, The British Horse Society, World Horse Welfare, SEIB, The Horse Trust, Equus magazine and Horse and Hound magazine. The demographics of the study respondents are presented first in the results to provide an overview of the study population. In the online questionnaire, this was the final part completed by participants.

### Horse owner demographics

The modal age category of participants was between 40–44 years old (13%, n = 178/1424). The study population composition was 75% UK based (n = 1059/1415), and 20% from the USA or Canada (n = 277/1415) and 5% from the rest of the world (Supplementary Item 2). The majority (98%) of participants were female (n = 1356/1387). Half of the participants had no formal equine qualifications (52%, n = 710/1359), whilst the other 50% varied in the formal equine qualifications they held, with the most common being British Horse Society qualifications (17%; n = 232/1359) and Pony Club tests (10%; n = 135/1359).

Information about participant’s experience of keeping horses, use of their horse, contact time, yard management and their opinion of their relationship with their veterinarian is presented in Supplementary Item 2.

### Horse owners’ assessment and understanding of the ‘normal’ horse

The first section of the survey evaluated participants’ knowledge of the normal, healthy horse. Participants were asked about the normal health parameters they believed they could measure accurately in their horse. The majority of participants believed they could accurately measure mucous membrane colour (80%; n = 1238/1547), rectal temperature (73%; n = 1133/1547), borborygmi (65%; n = 1007/1547), respiration rate (62%; n = 966/1547), skin tenting (54%; n = 841/1547) and heart rate (53%; n = 823/1547); 7% (n = 106/1547) of participants did not feel they could measure any of the given parameters accurately.

**Figure 1:**
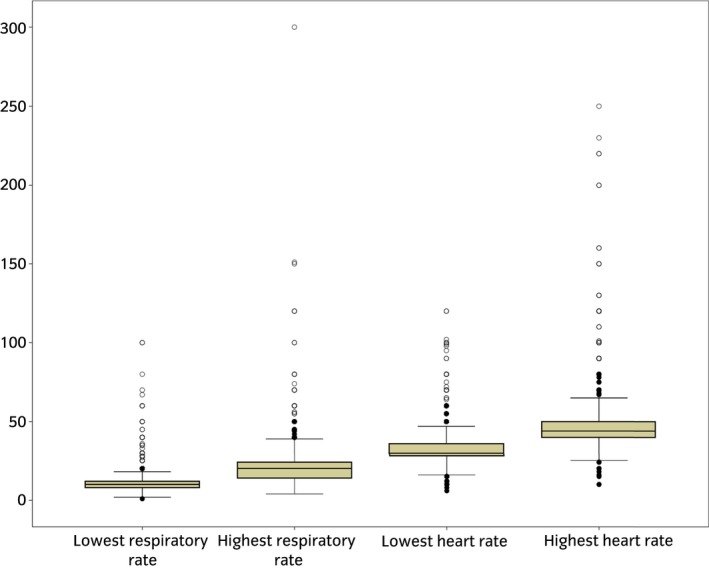
Boxplot of the responses of participants (n = 1540) when asked to provide the low and high values for the normal reference range of heart rate and respiratory rate in an online surgery of horse owner's knowledge and understanding of colic. Circles represent responses between 1.5 and 3 times the interquartile range, and asterisks greater than three times the interquartile range.

Participants were asked to give the lowest and highest normal values for heart rate, respiratory rate and rectal temperature. Their responses were compared with the defined reference ranges to determine if any values given (both lowest and highest values nominated by participants) fell outside this normal range. When asked about normal heart rate, 45% (n = 693/1540) of answers were within the reference range (28–44 beats per min), 27% (n = 416/1540) were outside the reference range and 28% (n = 431/1540) of participants were unsure and did not provide values (Fig 1). Values for normal heart rate provided by owners ranged between 6 and 250 beats per min. For respiratory rate, 45% (n = 694/1541) of participants gave values that fell within the reference range (8–16 breaths per min), 25% (n = 385/1541) of answers were outside the reference range and 30% of participants (n = 462/1541) were unsure and did not provide an answer (Fig 1). Values provided ranged between 2 and 300 breaths per minute. When asked the horse’s rectal temperature, 67% (n = 1028/1534) of responses were within the normal reference range (36.5–39.0°C), 12% (n = 184/1534) were outside and 21% (n = 322/1534) of participants were unsure and did not provide a value. The values provided ranged between 16 and 80°C. There was a significant association between knowledge of normal heart rate (P<0.01), respiratory rate (P<0.01) and rectal temperature (P<0.01) and participants with formal equine qualifications (specifically those who held equine‐specific college qualifications as a minimum). Age, experience and contact time had no statistically significant relationship with participants’ knowledge of normal values.

### Horse owners’ assessment and understanding of colic in the horse

This section of the survey evaluated how participants would assess and respond to signs of colic in their horse. The first questions in this section asked participants how they would respond if they observed specific changes in their horse(s) if all other parameters remained ‘normal’. The ‘changes’ were split into three sections: changes to a horse’s defaecation, behavioural changes and clinical changes. Data on responses to changes in defaecation and clinical changes are presented in Supplementary Item 3.

In response to behavioural changes, the majority of participants would monitor/observe horses that were quiet or dull (83%; n = 1280/1552), fence or box walking (79%; n = 1220/1552), weight shifting (70%; n = 1075/1552) or pawing the ground (60%; n = 914/1552). The majority of participants would call the veterinarian for horses that were rolling for an extended period/multiple times (90%; n = 1390/1552), lying down and getting up restlessly (88%; n = 1358/1552), lying down and getting up multiple times (65%; n = 1002/1552) or kicking at the abdomen (64%; n = 999/1552) (Supplementary Item 4). There were mixed responses to the behaviour changes of flank‐watching and inappetence: 50% (n = 770) would call the veterinarian for horses that were flank‐watching, and 42% (n = 648) would call the veterinarian for inappetent horses. Most participants selected the response that they would not call a veterinarian, if the horse was lying quietly or rolling for less than five minutes (Supplementary Item 4). In the scenario where the horse was exhibiting box walking behaviour, the decision to monitor a horse or call a veterinarian was significantly associated with age (P<0.01) or experience of a colic case in the previous year (P<0.01). In the scenario where the horse was pawing at the floor, the decision to monitor or call a veterinarian was significantly associated with previous experience of a critical case (P = 0.025). For a horse that had been rolling for more than five minutes/multiple times the decision‐making behaviour was significantly associated with owners holding qualifications equivalent to or higher than college level (P = 0.048). Finally, where a horse was lying down and getting up multiple times, there was a significant association with previous experience of a critical case of colic (P = 0.043) (Supplementary Item 4).

Participants were then asked for their definition of the term ‘colic’ using a free text response. There were 1393 free text responses, which were reviewed and categorised into 42 different themes. Pain attributed to a variety of sources was commonly mentioned, as was a problem associated with different abdominal organs, and specific conditions. An appropriate definition/explanation of colic, relating to abdominal pain with a range of different underlying causes, was given by 20% (n = 284/1393) of participants.

The final question in this section asked owners what they would assess in their horse, prior to contacting anyone else, if they thought it had colic. In a horse with suspected colic, the majority stated that they would assess defecation (76%; n = 1131/1486), borborygmi (75%; n = 1113/1486), respiration rate (65%; n = 967/1486) and heart or pulse rate (54%; n = 797/1486). A small proportion of participants (18%; n = 268/1486) stated that they would not check anything themselves and would call a veterinarian immediately on identifying signs of colic.

### Use of information and resources

Participants were asked to select all sources of information that they would use to find out more about colic from a predefined list. Most participants would ask veterinarians (83%; n = 1233/1486) or use the internet (73%; n = 1151/1486), followed by books (50%; n = 740/1486), and other resources (36% and fewer) (Supplementary Item 5). The two main sources of information nominated by the participants as contributing to their knowledge were personal experiences (86%; n = 1271/1482) and conversing with veterinarians (73%; n = 1088/1482).

Participants were asked where they thought the current gaps were in knowledge and information on equine colic, and how they would like information delivered and disseminated, using a free text response format. There were 940 responses. Twenty‐two themes were identified for gaps in knowledge, predominantly around the presentation and recognition of colic, and different causes of colic (Supplementary Item 6). Thirty‐four different preferred dissemination methods were identified by participants, with the most common methods being via the internet or through leaflets and fact sheets.

### Personal experiences of colic

There was a wide distribution of experience of colic, ranging from none to over 30 cases experienced. The modal category was three or four episodes (20%; n = 294/1464).

**Figure 2:**
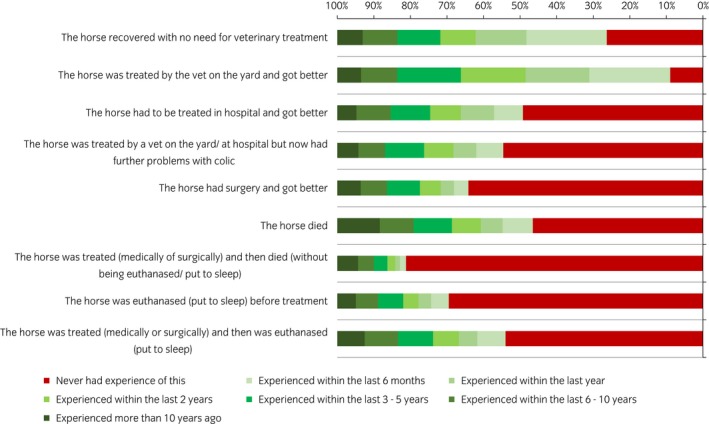
Specific types of colic experience of participants (n = 1433) in an online survey of horse owners’ knowledge and understanding of colic.

Owners’ experience of the types of colic ranged from horses recovering without needing veterinary treatment, through to critical cases requiring surgery, euthanasia or death of horses (Fig 2). Most participants reported that they had experience of a horse being treated by a veterinarian on a yard and recovering (91%; n = 1228/1433), and a horse recovering without needing veterinary treatment (74%; n = 954/1433). Most participants also had experience of a horse dying from colic (54%; n = 648/1433) or requiring hospitalisation for treatment (51%; n = 611/1433).

### Attitudes to decision‐making for horses with colic

Participants were asked to select the description that reflected their ability to recognise different cases of colic. The majority (61%; n = 916/1490) were confident that they would recognise most cases of colic unless it was an unusual presentation or unfamiliar horse, 29% (n = 436/1490) believed they would recognise some but not all cases or severities, and 7% (n = 99/1490) of participants were confident that they could recognise every case of colic including colic in different horses and severities. Participant’s confidence in their colic recognition was significantly associated with experience of a critical case (P<0.01), colic experience within the previous year (P<0.01), length of horse ownership (P<0.01) and equine qualifications at college level or higher (P<0.01), but was not significantly associated with contact time (P = 0.08) or owner age (P = 0.3).

**Figure 3:**
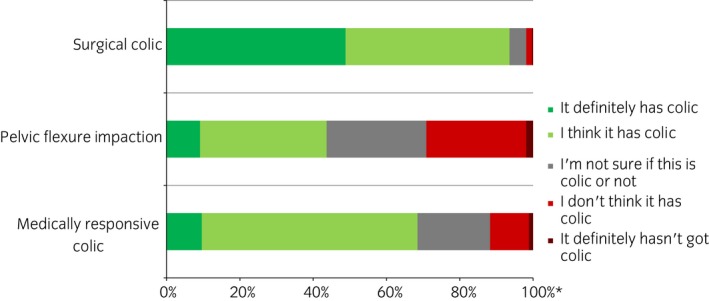
Participants’ certainty that a horse had colic or not when provided with different scenarios in an online survey of horse owners’ knowledge and understanding of colic (n = 1427–1434). Participants were provided with three scenarios (based on presentation and signs that would/might be seen with a surgical colic, pelvic flexure impaction and a medically responsive colic) and they were asked how likely they thought it was that the horse in the scenario had colic. The red area on the bar indicates that the participant thought that the horse did not have colic, whilst the green that they thought it did have colic. The darker shades demonstrate a greater certainty. *Expressed as a percentage of the number of respondents for each case outcome.

When asked about three different colic scenarios, for the surgical colic scenario, 94% (n = 1340/1433) of participants thought that the horse had colic. For the medical colic scenario, 68% (n = 982/1434) thought that the horse had colic, while 20% (n = 284/1434) were unable to distinguish whether the horse had colic or not and the remaining 12% (n = 168/1434) did not think the horse had colic. For the colic caused by a pelvic flexure impaction, 44% (n = 626/1433) identified that the horse had colic, while 27% (n = 390/1433) were unable to distinguish whether the horse had colic or not and the remaining 29% (n = 417/1433) did not think the horse had colic (Fig 3). Almost half (49%; n = 700/1433) of participants stated they were definitely sure that the horse in the surgical colic scenario had colic, whereas fewer participants were so definitive about the horses having colic in the medical scenario (10%; n = 138/1434) and the pelvic flexure impaction scenario (9%; n = 131/1433) (Fig 3).

## Discussion

This study describes the perspective of horse owners’ knowledge, understanding and experience of colic, and their attitudes to recognising and responding to signs of colic. There were a number of areas of incongruence, including owners’ high rating of their ability to recognise colic, compared with their responses when presented with different case scenarios, and the clinical parameters that they said they would assess compared with their knowledge of normal values for these parameters. Attitudes to calling a veterinarian in response to different behavioural signs of colic, showed significant variation, suggesting that there is no clear consensus on this essential aspect of decision‐making. Recent experience of colic, experience of critical cases of colic, and having a further education qualification in equine studies were all significantly associated with key components of colic decision‐making and require further exploration through qualitative studies. The study clearly identified the need for further resources to support decision‐making in horse owner.

There are several potential biases that may affect the study. The survey was distributed online and through social media channels, which will result in selection bias for participants. The majority of the respondents were UK‐based, but there were no differences in responses between UK and non‐UK participants when compared during preliminary data analysis (data not shown). The demographics of the study population were similar to other studies in the literature 9, 10, 11 with an even spread of ages and a strong female bias to the study population. The total sample population that the survey reached was unknown so an overall response rate could not be calculated, but the study had a high completion rate (85%). Self‐selection bias is likely, as owners with previous experience, or an interest in colic, are more likely to participate. The data on owners’ experience of colic are, therefore, unlikely to be representative of the wider population. The study was conducted in 2014, and may not represent current knowledge and opinions, especially as this study lead to a subsequent educational campaign in the UK (www.bhs.org.uk/colic). Finally, this questionnaire, similar to others, reflects opinions and may be subject to recall bias; an observational study to assess actual responses in different situations would be required to validate findings.

The most important findings relate to the horse owners’ opinions and attitudes, including their approaches to decision‐making, and any knowledge gaps that may affect how they recognise and respond to signs of colic. Over half the participants believed they could accurately measure a number of clinical parameters in the normal horse, including mucous colour (80%), rectal temperature (73%), gut sounds (65%), respiration rate (62%) and heart rate (53%). A significant number also stated that if their horse had colic, they would assess some clinical parameters, specifically gut sounds (75%), respiration rate (65%) and heart rate (54%) before they contacted anyone else. These parameters can be important indicators of the severity of the condition 12, 13, 14. The findings of this study demonstrate owners’ willingness to be actively involved in the assessment of their horse’s health, but that there are issues with how their assessments may be interpreted. Less than 50% of participants gave answers within the normal range for heart rate and respiratory rate, and some of the values given were markedly outside of the normal ranges. A number of participants were clearly aware of their lack of knowledge of normal values, but others were not aware that their knowledge was inaccurate. It is also likely that this study overestimated knowledge of normal parameters, as even though participants were asked not to look up the information, some may have done this; a true assessment would require a test‐like situation with no access to resources. The parameters that were investigated in this study (accurate assessment of heart rate, respiratory rate and rectal temperature) can help owners recognise a range of different diseases (for example using assessing rectal temperature or respiratory rate to monitor for infectious respiratory disease). This lack of knowledge about normal values, therefore, has wider implications than the colic focus of this study. Improving horse owners’ knowledge of normal parameters will bring benefit across a wide range of diseases.

The questions on how participants would respond to behavioural and clinical changes in their horse were constructed to explore decision‐making in horses with colic, based on previous research 9. The clinical signs that would prompt seeking veterinary assistance (distended abdomen, getting up and down, kicking at the abdomen and a horse that was thrashing around) were consistent with the study by Scantlebury *et al.* 9, adding to the evidence on key signs that influence decision‐making. The current study also identified several signs where many owners would not call a veterinarian. Less than 50% of the owners would call the veterinarian for a horse that was quiet/dull, fence/box walking, weight shifting, pawing the ground, flank‐watching, inappetent, rolling for less than five minutes, or lying down quietly; they would choose instead to monitor, observe or not be concerned. These are non‐specific mild signs, which may be seen in normal horses, but are also potential signs of colic. They may be the only signs in less severe types of colic, such as large intestinal impactions 15, or early signs of other severe conditions, such as colitis or peritonitis. Again, this highlights potential issues around decision‐making for horses showing less severe or non‐specific signs of colic.

Participants were asked to provide a definition of the term colic, with approximately a fifth providing an appropriate answer. A common theme that was observed throughout the free‐text comments was specific gastrointestinal causes of colic. The understanding of colic being a gastrointestinal malfunction is consistent with the study by Scantlebury *et al* 9. Although colic is primarily caused by gastrointestinal issues, it refers to abdominal pain, caused by diseases of any abdominal organs, and there may be a wide range of underlying causes 12. This misconception of a single disease may again relate back to issues around recognising different signs of colic. A key finding of this study is the need for clarity of information about colic for owners, including what colic is, the range of different signs that may be shown, and how to respond to these signs.

Participants’ had a high confidence in their ability to recognise different types of colic, which was not reflected in the scenario responses. In the scenarios, participants were much better at recognising a more severe case of colic, and very few were confident in recognising the cases with milder signs. This suggests that participants may be less accurate at recognising a horse with colic than they believe. Many horse owners wrote in the free text comments that they would be surer of their decision if they had been provided with a video or a ‘real life’ situation in their own horse. The case scenarios were based on the most likely presentation for different conditions; however, clinical presentations can vary with some gastrointestinal and non‐gastrointestinal causes having similar presentations which introduces potential error or bias to this type of question. Diagnosis can be challenging for both veterinarians and owners. This study has, however, highlighted variations in horse owners’ recognition and confidence in decision‐making when presented with different scenarios, especially those with less severe clinical signs. Whilst rapid decision‐making is essential in horses with severe lesions, these represent a relatively small proportion of cases, and even the critical cases may present with less marked or obvious clinical signs. The response to the scenarios agreed with participants’ attitudes to behavioural changes, where signs such as being quiet or dull, lying down or inappetant often did not trigger a response to call the veterinarian.

The approaches used by horse owners to find information has been previously investigated 16, 17. In the current study, the majority of participants’ knowledge was obtained from personal experience and information from veterinarians. Personal experience was also identified by Scantlebury *et al*. 9 as an important factor in decision‐making, and in the current study both experience of critical cases and recent colic experience (in the previous year) significantly affected owners’ confidence in colic recognition. The over‐arching finding from this study was the need for further education and resources for horse owners about colic, both from the knowledge gaps and issues identified within the questionnaire, and from the many (n = 940) free text comments and suggestions for further resources. Following the work described in this manuscript, the results from this survey were presented in stakeholder workshops, using a co‐production methodology involving horse owners 18, to develop an educational campaign for horse owners on colic (http://www.bhs.org.uk/colic). The information leaflets and the methods of dissemination used for the campaign were based on the suggestions and ranking of themes on knowledge gaps and use of resources from this study. This included a focus on recognising less severe signs of colic and calling the veterinarian to ask for advice as early as possible.

This is the first study describing responses from horse owners on their knowledge, attitude and practices towards a common and critical condition in the horse. It is pivotal in informing how we develop support mechanisms and educational resources to enable owners to make timely and appropriate responses to emergency diseases in their animals. As a condition, colic is poorly understood by horse owners, with confusion and knowledge gaps around what ‘colic’ is, the different signs that may be shown, and how to assess and respond to them. The disparity between horse owners’ confidence and ability to recognise colic is concerning, as is the variation in response to different behavioural signs; the common types of colic which present with less severe signs represent a challenge. An owner focussed educational campaign is necessary in order to inform owners about colic, help them recognise the different signs, and respond appropriately to seek intervention by veterinary surgeons.

## Authors’ declaration of interests

No competing interests have been declared.

## Ethical animal research

The study was reviewed and approved by the School of Veterinary Medicine and Science Ethics Committee, University of Nottingham.

## Owner informed consent

All participants gave informed consent to participate in this study.

## **Data** **accessibility** **statement**

The data that support the findings of this study are available from the corresponding author upon reasonable request.

## Source of funding

Adelle Bowden's PhD studentship was supported by funding from the School of Veterinary Medicine and Science, University of Nottingham.

## Authorship

A. Bowden was the main researcher, with primary responsibility for data collection, analysis and preparation of the final manuscript, and major contribution to study design. J. Burford had primary responsibility for the statistical analysis methodology. J. Burford, M. Brennan, G. England and S. Freeman all contributed to the study design and methodology, data interpretation and preparation of the manuscript. A. Bowden and J. Burford had full access to all data in the study and take responsibility for the integrity and accuracy for the data analysis. All authors have reviewed and approved the final manuscript.

## Supporting information


**Supplementary item 1:** Questionnaire used for an online survey for horse owners about recognising colic in the horse.

**Supplementary item 2:** Details of demographic information and opinion of relationship with their veterinarian for participants in an online survey of horse owners’ knowledge and understanding of colic (n = 1420).

**Supplementary item 3:** Participants responses to changes in horses’ defaecation and clinical changes in an online survey of horse owners’ knowledge and understanding of colic (n = 1554).

**Supplementary item 4:** Horse owner’s opinions (n = 1552) of how they would react to specific, isolated changes in and behaviour in their horse in an online survey of horse owners’ knowledge and understanding of colic.

**Supplementary item 5:** Areas where information is lacking on colic in the horse, identified by horse owners (n = 940) in their horse in an online survey of horse owners’ knowledge and understanding of colic.

**Supplementary item 6:** Use of information and resources by participants in an online survey of horse owners’ knowledge and understanding of colic (n = 1486).

## Manufacturers' addresses

1 SurveyMonkey, California, USA.

2 IBM Corporation, Armonk, New York, USA.
