# Supplementary material for: Horse owners’ knowledge, and opinions on recognising colic in the horse
Source: Equine Vet J. 2019 Sep 23;52(2):262–7. doi: 10.1111/evj.13173 (PMC7027804; doi:10.1111/evj.13173)
Supplement: Supplementary file 1 — Supplementary item 1: Questionnaire used for an online survey for horse owners about recognising colic in the horse. [file EVJ-52-262-s001.pdf]

The 'normal' horse

This questionnaire is not a test. Please answer the following questions as honestly as possible, as this will provide us with the most beneficial information. We are not judging responses. There are no right or wrong answers. We are most interested in which aspects people find difficult, so we can try and develop guidance to help with this. Please do not use books/ phones/ internet to assist you in answering questions. Many thanks.

3. What do you think the normal heart rate range is for an average horse at rest?

(Heart rate at rest is the number of beats per minute when not under stress or exercise.
Range is defined as the lowest and highest values considered to be normal.
An average horse is 500kg, 16hh fit general purpose horse.)

If you don't know please insert a "?" in the answer field.

Lowest value

Highest value

4. What do you think the normal respiration (breathing) rate range is for an average horse at rest?

(Respiration rate at rest is the number of breaths per minute when not under stress or exercise.
Range is defined as the lowest and highest values considered to be normal.
An average horse is 500kg, 16hh fit general purpose horse.)

If you don't know please insert a "?" in the answer field.

Lowest value

Highest value

5. What do you think the normal temperature range is for an average horse at rest?
Please indicate the units used (e.g. celsius/ farenheit) if you know them.

(Range is defined as the lowest and highest values considered to be normal.
An average horse is 500kg, 16hh fit general purpose horse.)

If you don't know please insert a "?" in the answer field.

Lowest value

Highest value

Units

6. What do you think normal gut sounds are for an average horse at rest?

(Gut sounds are the sounds that a horse's intestines make that can be heard over the abdomen with a stethoscope or by placing your ear to the flank.
An average horse is 500kg, 16hh fit general purpose horse.)

- ◯ No sounds present at all
- ◯ Some tinkling and occasional quiet gurgles
- ◯ Some tinkling and regular gurgles, some quite loud, lasting around 3 seconds
- ◯ Constant tinkling sounds and gurgles, very loud and can be heard very easily without stethoscope
- ◯ Don't know

7. Which of the following do you feel confident that you can measure accurately?
Please tick ALL that apply

- ☐ Heart/ pulse rate
- ☐ Respiration rate
- ☐ Temperature
- ☐ Gut sounds
- ☐ Gum colour
- ☐ Capillary refill time (CRT)
- ☐ Skin tenting
- ☐ Digital pulses
- ☐ None of the above

8. How many droppings would you expect an average horse to pass in a 24 hour period?

(A dropping is a pile of nuggets which is normal for that horse or appropriate for the size and breed of horse.
An average horse is 500kg, 16hh fit general purpose horse.)

If you don't know please insert a "?" in the answer field.

From ____________________

To ____________________

9. We would like some information on how you decide what is normal for your horse.

How would you react to the following changes to a horse's droppings? Please interpret this as if it were the only change to your horse.

	Not concerned	Monitor or observe	Look on the internet/ other resource	Call a friend/ other person	Call the vet	Wouldn't know how to interpret this
Mucous covered droppings (normal consistency)	○	○	○	○	○	○
Blood in droppings (normal consistency)	○	○	○	○	○	○
Worms in droppings (normal consistency)	○	○	○	○	○	○
Change in colour of droppings (normal consistency)	○	○	○	○	○	○
Drier droppings than normal	○	○	○	○	○	○
Wetter droppings than normal (cow pat consistency - still have some form)	○	○	○	○	○	○
Very wet dropping (watery consistency, may be projectile, no form to dropping)	○	○	○	○	○	○
Fewer droppings than normal	○	○	○	○	○	○
More droppings than normal	○	○	○	○	○	○

10. Please indicate for each of these behavioural changes whether you would consider these abnormal and what action you would take. Please assume for each 'change' that this is the only one the horse is showing.

	Not concerned	Monitor or observe	Look on the internet/ other resource	Call a friend/ other person	Call the vet	Wouldn't know how to interpret this sign
Horse is flank watching (turning head towards belly)	○	○	○	○	○	○
Horse is lying down quietly	○	○	○	○	○	○
Horse is lying down and getting up (2-3 times in 30 minute period)	○	○	○	○	○	○
Horse restlessly lying down and getting up (4+ times in 30 minute period)	○	○	○	○	○	○
Horse is rolling (for less than 5 minutes)	○	○	○	○	○	○
Horse rolling for more than 5 minutes or rolling multiple times (4+) in a 30 minute period	○	○	○	○	○	○
Horse pawing at floor (not in response to food or other stimulus)	○	○	○	○	○	○
Horse kicking at belly (no obvious external stimulus e.g. flies)	○	○	○	○	○	○
Horse not eating/ gone off food	○	○	○	○	○	○
Fence/ box walking	○	○	○	○	○	○
Quiet or depressed	○	○	○	○	○	○
Shifting from one foot to the other	○	○	○	○	○	○

11. Please indicate for each of these clinical changes whether you would consider these abnormal and what action you would take. Please assume for each 'change' that this is the only one the horse is showing

	Not concerned	Monitor or observe	Look on the internet/ other resource	Call a friend/ other person	Call the vet	Wouldn't know how to interpret this sign
Unexpected sweating	◯	◯	◯	◯	◯	◯
Increase in respiration (breathing) rate	◯	◯	◯	◯	◯	◯
Decrease in respiration (breathing) rate	◯	◯	◯	◯	◯	◯
Increase in heart rate	◯	◯	◯	◯	◯	◯
Decrease in heart rate	◯	◯	◯	◯	◯	◯
Increase in body temperature	◯	◯	◯	◯	◯	◯
Decrease in body temperature	◯	◯	◯	◯	◯	◯
Straining to urinate/ defecate	◯	◯	◯	◯	◯	◯
Distended belly	◯	◯	◯	◯	◯	◯
Diarrhoea	◯	◯	◯	◯	◯	◯
Weight loss	◯	◯	◯	◯	◯	◯

12. Please use this box to add any further comments on any of the questions so far if you wish. A text box will be available for comments at the end of each section.

Colic in the horse

13. What do you think the term 'colic' means?

14. What would you check on your horse if you thought it had colic before contacting anyone else? Please tick ALL that apply.

- ☐ Heart/ pulse rate
- ☐ Respiration (breathing) rate
- ☐ Rectal temperature
- ☐ Ear temperature
- ☐ Hoof temperature
- ☐ Nasal discharge
- ☐ Ocular (eye) discharge
- ☐ Lymph nodes (glands)
- ☐ Gut sounds (listening with stethoscope to abdomen)
- ☐ Gum (mucous membrane) colour
- ☐ Capillary refill time (time for colour to return after pressing on gums)
- ☐ Digital pulses (pulses felt near fetlocks)
- ☐ Skin tenting (recoil of skin when pulled)
- ☐ I wouldn't check anything myself, I would call someone more experienced (not a vet) to check the horse over
- ☐ I wouldn't check anything myself, I would call a vet and get them to come and check the horse over
- ☐ Urination
- ☐ Defecation (faeces)

Other (please specify)

15. How confident would you be that you could recognise colic in a horse?

- ◯ I would recognise every case in different horses with different presentations and severities
- ◯ I would recognise most cases unless it was a very odd presentation or an unfamiliar horse
- ◯ I could recognise it in some cases but not in all cases/ all severities
- ◯ I wouldn't recognise it unless very severe/ obvious
- ◯ I wouldn't recognise it at all

16. If you were NOT in an emergency situation and wanted to find out more about how to recognise and deal with colic, where would/do you go for more information?
Please tick ALL that apply.

- ☐ Ask a vet
- ☐ Ask another professional (farrier, physio etc)
- ☐ Ask friends/ family
- ☐ Ask yard owner/ trainer
- ☐ Look on the internet
- ☐ Search phone app
- ☐ Look at a poster
- ☐ Look at a factsheet
- ☐ Look in a book
- ☐ Look in magazines
- ☐ Look at forums
- ☐ Look at Pony Club information
- ☐ Look at British Horse Society (BHS) information
- ☐ Look to another professional organisation for information

Other (please specify)

17. It would be very helpful if you could give us more specific details of where you would go for more information in the box below.
(e.g. which people/ magazines/ forms etc.)

18. Thinking about your current knowledge of colic in the horse, where did this information/ knowledge come from?

Please tick ALL that apply.

- [ ] Reading a factsheet
- [ ] Talking to a vet
- [ ] Talking to another professional (farrier, physio etc)
- [ ] Talking to friends/ family
- [ ] Talking to your yard owner/ trainer
- [ ] Personal experience
- [ ] Looking at the internet
- [ ] Using a phone app
- [ ] Reading a poster
- [ ] Reading books
- [ ] Reading magazines
- [ ] Using forums
- [ ] Looking at/ using Pony Club information
- [ ] Looking at/ using British Horse Society (BHS) information
- [ ] Information/ course from another professional organisation
- [ ] Not sure

Other (please specify)

19. Again, it would be very helpful if you could give us more specific details of where you got your knowledge from in the box below.
(e.g. which people/ magazines/ forms etc.)

20. We are particularly interested in where the gaps are in the information/ education that is currently available on equine colic for horse owners.

Please could you tell us which areas of equine colic you would like more information on?

How you would like this information made available to you?

Personal experiences

This section is designed to help us understand the experiences of different horse owners. Again, this is not a test so please answer as honestly as possible.

21. Thinking about colic in the horse, how many different episodes have you personally seen?

- ○ None
- ○ 1 - 2
- ○ 3 - 4
- ○ 5 - 7
- ○ 8 - 10
- ○ 11 - 20
- ○ 21 - 30
- ○ 30+

22. If you have seen horses with colic, please can you give us more details about any personal experiences that you have had with horses that you own or look after.

	Never had experience of this	Within the last 6 months	Within the last year	Within the last 2 years	Within the last 3 - 5 years	Within the last 6 - 10 years	Experienced more than 10 years ago
The horse recovered with no need for veterinary treatment	○	○	○	○	○	○	○
The horse was treated by the vet on the yard and got better	○	○	○	○	○	○	○
The horse had to be treated in hospital and got better	○	○	○	○	○	○	○
The horse was treated by a vet on the yard/ at hospital but now had further problems with colic	○	○	○	○	○	○	○
The horse had surgery and got better	○	○	○	○	○	○	○
The horse died	○	○	○	○	○	○	○
The horse was treated (medically of surgically) and then died (without being euthanased/ put to sleep)	○	○	○	○	○	○	○
The horse was euthanased (put to sleep) before treatment	○	○	○	○	○	○	○
The horse was treated (medically or surgically) and then was euthanased (put to sleep)	○	○	○	○	○	○	○

23. How often do you read/ research about colic (including reading books / magazines, looking on the internet, talking to people etc.)?

- ○ Once a week
- ○ Once a month
- ○ Once every 6 months
- ○ Once a year
- ○ Once every 2 - 3 years
- ○ Very rarely (less than every 3 years)
- ○ Never

Other (please specify)

24. Please use this box for any comments you have about any questions in this section, if you wish.

Diagnosis scenarios

This section is about trying to decide whether a horse has colic or not. We have given you a series of 'scenarios' to look at, and decide if you think the horse in that scenario has colic or not.

Please remember that there are no right or wrong answers, and we are most interested in which aspects people find difficult, so we can try and develop guidance to help with this. Therefore, please answer the questions as honestly as possible.
Many thanks.

25. Scenario 1:

You are out hunting with your friend. She is on her new 6 year old thoroughbred horse that she has owned for 2 weeks. This is the first hunt with her. Two hours into the hunt the horse pulls up and will not move. The horse is sweating and shaking and will not move from where it is stood. There are no obvious cuts or swellings on any of the legs. The horse has a heart rate of 68 beats per minute and a respiratory rate of 28 breaths per minute. The gut sounds are normal. The horse urinates and it looks a dark brownish colour.

How likely do you think it is that this horse has colic?

- ◯ It definitely has colic
- ◯ I think it has colic
- ◯ I'm not sure if this is colic or not
- ◯ I don't think it has colic
- ◯ It definitely hasn't got colic

26. Scenario 2:

It's late spring. You go to get your 7 year old TBx general purpose horse in from the field and find he is covered in mud and sweating. He also seems to be breathing a bit faster than normal. There are 4 other horses in the field which are eating normally and look OK. He is passing quite a lot of wind, has a heart rate of 44, respiratory rate of 24 and temperature of 38.2 degrees Celsius (100.8 Fahrenheit). His gut sounds are increased. He passes a normal looking dropping on the way back from the field, but is quite restless in his stable.

How likely do you think it is that this horse has colic?

- ◯ It definitely has colic
- ◯ I think it has colic
- ◯ I'm not sure if this is colic or not
- ◯ I don't think it has colic
- ◯ It definitely hasn't got colic

27. Scenario 3:

You are tending to your 12 year old intermediate event horse when you notice he seems quieter than usual. He has been on box rest since he got a nasty cut at a competition that needed stitches 8 days ago. Prior to this he spent equal time housed and at pasture, and was exercised twice a day. He hasn't eaten all of his haynet and you've noticed he hasn't drunk as much as normal over the past 3 - 4 days. He keeps stretching and turning his head around, to look at the flank of his injured leg. His heart rate is 28, respiratory rate is 12 and temperature is 37.5 degrees Celsius (99.5 Fahrenheit).

How likely do you think it is that this horse has colic?

- It definitely has colic
- I think it has colic
- I'm not sure if this is colic or not
- I don't think it has colic
- It definitely hasn't got colic

28. Scenario 4:

Another owner on your livery yard is distressed when tending her horse first thing in the morning. Her 15 year old arab gelding has a cut eye that is very swollen. He is normally highly strung and on closer inspection there are some other abrasions to both forelimbs, around the head and over the hip bones. He has a swollen right fore leg, is very dirty and has a lot of bedding stuck to his coat. He was last checked about 8 hours ago by the yard owner, who gave him an extra haynet, when he was fine. He's now sweaty and unsettled and is walking around the box and kicking at his belly. He has left half of his haynet and his bed is a mess which is unusual for him. Someone on the yard is a vet nurse and takes his heart rate which is 80 beats per minute, respiratory rate is 28 breaths per minute and gums are pale with a capillary refill time of 2.5 seconds.

How likely do you think it is that this horse has colic?

- It definitely has colic
- I think it has colic
- I'm not sure if this is colic or not
- I don't think it has colic
- It definitely hasn't got colic

29. Scenario 5:

An 18 year old pony which has been diagnosed with a condition called Cushings disease is lying down in your neighbour's field and refusing to get up. He is on pills for his Cushings and the vet has said it's under control. It is March and because it has been very wet, he's been in the stable for a few weeks. He has now been on grass for 3 days, with access to shelter. He was fine at 8am this morning (8 hours ago), and ate his feed and medication as normal. Apart from refusing to get up, he seems quite weak and he is a bit sweaty under his mane but there are no obvious cuts on him. He has a heart rate of 44 beats per minute and a respiratory rate of 24 breaths per minute. He has normal gut sounds

How likely do you think it is that this horse has colic?

- ◯ It definitely has colic
- ◯ I think it has colic
- ◯ I'm not sure if this is colic or not
- ◯ I don't think it has colic
- ◯ It definitely hasn't got colic

30. Scenario 6:

Your friend asks you to have a look at her 6 year old Welsh cob as she thinks there is something wrong. She moved to your yard a couple of weeks ago so that you could go hacking together. The horse has been in the field with both of your horses (which are perfectly healthy) since it was wormed last week. The horse seems very quiet and you notice it is very sweaty with twitching muscles. The horse is standing very oddly with its 4 feet very close together and looks very weak as its eyes are half shut. It is not interested in eating, although it has a small amount of what looks like food at both nostrils.

How likely do you think it is that this horse has colic?

- ◯ It definitely has colic
- ◯ I think it has colic
- ◯ I'm not sure if this is colic or not
- ◯ I don't think it has colic
- ◯ It definitely hasn't got colic

31. Please feel free to make any comments regarding the scenario section, should you wish.

Demographics

Finally, could you please provide some information about yourself. This will be treated with the strictest confidence, and will be coded to provide anonymity.

32. What age category do you belong to?

- ○ Under 16
- ○ 16 - 24
- ○ 25 - 29
- ○ 30 - 34
- ○ 35 - 39
- ○ 40 - 44
- ○ 45 - 49
- ○ 50 - 54
- ○ 54 - 59
- ○ 60 - 64
- ○ 65+

33. Are you..

- ○ Male
- ○ Female

34. Approximately how long have you had horses for (owned/ loaned/ cared for)?

- ○ less than 6 months
- ○ 6 months - 1 year
- ○ 1 - 2 years
- ○ 3 - 5 years
- ○ 6 - 10 years
- ○ 11 - 20 years
- ○ 21 - 30 years
- ○ 31 - 40 years
- ○ 41+ years

35. How many horses do you have (own/ loan/ care for) at the moment?

Number of horses ______

36. What is the MAIN activity that you undertake with your horse(s)?

- Hacking
- Schooling
- Showjumping
- Eventing
- Dressage
- Showing
- Breaking and/or training
- Hunting
- Endurance
- Horseball
- Polo
- Racing/ Point to point
- Driving
- Retired/ companion horse
- Breeding

Other (please specify)

37. Where are you based?

- UK
- Ireland
- Europe
- USA
- Australia

Other (please specify)

38. Please nominate the highest level you compete your (top) horse at.

- I never compete
- I compete at local shows only
- I compete at unaffiliated shows nationally
- I compete at affiliated shows nationally
- I compete at affiliated shows internationally

Other (please specify)

39. What BEST describes the time you spend with your horse(s)?

- I spend all of my time with my horse(s) (At least 8 hours every day, including weekends)
- I spend most of my time with my horse(s) (between 4 and 7 hours every day, including weekends)
- I spend a lot of time with my horse(s) (between 2 and 3 hours each day on weekdays and more at weekends)
- I spend a fair amount of time with my horse(s) (up to 2 hours each day in the week and more at weekends)
- I spend time with my horse at weekends only
- I spend time with my horse a couple of times a month
- I spend time with my horse 3 - 4 times a year
- I don't really spend time with my horse

40. What is the typical management of your horse(s)?

- Kept at grass
- Full DIY (kept at home)
- Full DIY (kept at yard)
- Assisted DIY
- Working livery
- Full livery
- Competition livery

Other (please specify)

41. Do you have any formal equine qualifications?
(If multiple, please choose the highest level of qualification)

- ○ No formal qualification
- ○ Pony club tests
- ○ College course (NVQ, National Diploma etc.)
- ○ BHS stages 1 or 2
- ○ BHS stage 3 or AI
- ○ BHS qualifications from BHSAI to BHSII
- ○ BHS qualifications greater than BHSII
- ○ Undergraduate degree
- ○ Postgraduate qualifications (Masters/ PhD etc)
- ○ Veterinary nursing qualifications
- ○ Veterinary medicine qualifications

Other (please specify)

42. Please choose what best describes your approach to calling a vet to your horse(s).

(not including routine calls such as vaccinations and dental work)

- ○ I call the vet out too often but would rather check there is nothing wrong
- ○ I always call the vet when needed and know when they're not needed
- ○ Generally, I always call the vet at the right time
- ○ I'm not sure that I call the vet at the right time all of the time
- ○ I don't know when I should call a vet out or not
- ○ There have been quite a few times the vet should have been called sooner

Other (please specify)

43. How would you describe your relationship with your vet practice?
Please tick ALL that apply

- [ ] You get on very well with the vets and can ring them directly for advice
- [ ] You get on well with the vets and would have them for calls but wouldn't ring them directly for advice
- [ ] You don't really know the vets, would call the practice if you needed advice/ an appointment
- [ ] You don't really get on well with the vets so would only contact the practice if absolutely necessary
- [ ] You always get a different vet, so don't feel very confident with them
- [ ] You think the vets are too young and don't feel very confident with them
- [ ] You feel embarrassed to ask the vets questions in case they aren't relevant
- [ ] You feel intimidated by the vets
- [ ] You use the practice website frequently
- [ ] You use the practice fact sheets frequently
- [ ] You would call more often but you worry about the cost of an unnecessary call out
- [ ] You would call more often but you don't want to call the vets out unnecessarily and waste their time

Other (please specify)

44. If you wish to make any further comments that you feel may be relevant to this study, please use the space provided below.
