# Supplementary material for: Horse owners’ knowledge, and opinions on recognising colic in the horse
Source: Equine Vet J. 2019 Sep 23;52(2):262–7. doi: 10.1111/evj.13173 (PMC7027804; doi:10.1111/evj.13173)
Supplement: Supplementary file 2 — Supplementary item 2: Details of demographic information and opinion of relationship with their veterinarian for participants in an online survey of horse owners’ knowledge and understanding of colic (n = 1420). [file EVJ-52-262-s002.pdf]

BEVA Equine Veterinary Journal

Supplementary Item 2: Details of demographic information and opinion of relationship with their vet for participants in an online survey of horse owners' knowledge and understanding of colic (n = 1,420).

This supplementary item provides additional demographic data on the participants, their equine experience and opinion of their relationship with their vet.

The modal age category of participants was between 40 to 44 years old (n = 178/1,424). The distribution of participants was between 9% and 15% for all age categories, except those under 16 years old and over 60 years old (1% and 5% respectively).

The geographical location of participants was: 75% from UK (n = 1059/1415), 20% from USA or Canada (n = 277/1415), 2% from Australia (n = 28/1415) and Europe (not UK or Ireland) (n = 24/1415), and less than 1% each from Ireland (n = 12/1415), South Africa (n = 6/1415), South America (n = 2/1415), Hong Kong (n = 1/1415) and Israel (n = 1/1415).

Over 90% of participants (n = 1,284/1,420) had kept horses for more than 5 years. The modal categories were 11 to 20 years (28%; n = 403/1,420), and 21 to 30 years (22%; n = 313/1420). The majority (77%) of participants kept between one and 4 horses (n = 1.098/1,421).

Horses were used for 15 different activities, most commonly hacking or leisure riding (39%; n = 549/1,331). The population of horse owners mostly competed at local shows (35%; n = 475/1,356) or never attended competitions with their horses (33%; n = 447/1,356).

The amount of contact time participants' (n = 1,415) spent with their horses varied, with most attending their horse on a daily basis for up to 3 hours on weekdays and more at weekends. The management of the horses was largely DIY livery (61%; n = 858/1,350) followed by grass livery (16%; n = 237/1,350).

When asked about their approach to calling a veterinarian, the majority of participants either felt they 'generally called the vet at the right time' (51%; n = 705/1,380) or 'always called the vet when they were needed and knew when they were not needed' (37%; n = 510/1,380). When asked about the veterinary-client relationship, 72% (n = 1,012/1,396) of participants felt they 'got on well with their vets and could ring them directly for advice'. There were 120 free text comments on the demographics and owner-veterinary relationship section. Twelve themes were identified from the comments: personal experiences and positive owner-veterinary relationships were most commonly mentioned.
