# Supplementary material for: Horse owners’ knowledge, and opinions on recognising colic in the horse
Source: Equine Vet J. 2019 Sep 23;52(2):262–7. doi: 10.1111/evj.13173 (PMC7027804; doi:10.1111/evj.13173)
Supplement: Supplementary file 3 — Supplementary item 3: Participants responses to changes in horses’ defaecation and clinical changes in an online survey of horse owners’ knowledge and understanding of colic (n = 1554). [file EVJ-52-262-s003.pdf]

BEVA Equine Veterinary Journal

Supplementary Item 3: Participants responses to changes in horse’s droppings and clinical changes in an online survey of horse owners’ knowledge and understanding of colic (n = 1,554).

This data is from the responses given by participants when asked how they would respond if they observed specific changes in their horse(s) if all other parameters remained ‘normal’. The changes related to changes in defaecation and other clinical changes. When asked about their response to changes in their horse’s defaecation, most participants would monitor/observe their horse for changes in: colour (75%; n = 1,163/1,554); consistency, if drier (85%; n = 1,317/1,554), or wetter but with some form remaining (85%; n = 1,314/1,554); number of faeces had increased (75%; n = 1,161/1,554) or decreased (85%; n = 1,310/1,554). The majority of participants would call a veterinarian: if there was blood present (85%; n = 1,317/1,554) or if the faeces were very wet/watery, possibly projectile with no form’ (52%; n = 800/1,554).

When asked about response to clinical changes, participants were divided between monitoring their horses and seeking veterinary advice. The majority would call the veterinarian if the horse was straining to urinate or defecate (78%, n = 1,203/1,550) or if the horse had a distended abdomen (64%, n = 984/1,550).
