# Supplementary material for: Horse owners’ knowledge, and opinions on recognising colic in the horse
Source: Equine Vet J. 2019 Sep 23;52(2):262–7. doi: 10.1111/evj.13173 (PMC7027804; doi:10.1111/evj.13173)
Supplement: Supplementary file 4 — Supplementary item 4: Horse owner’s opinions (n = 1552) of how they would react to specific, isolated changes in and behaviour in their horse in an online survey of horse owners’ knowledge and understanding of colic. [file EVJ-52-262-s004.pdf]

BEVA Equine Veterinary Journal

Supplementary Item 4: Horse owner's opinions (n = 1,552) of how they would react to specific, isolated changes in and behaviour in their horse in an online survey of horse owners' knowledge and understanding of colic.

This table provides the detail of the data for the questions on how owners would respond to changes in their horse's behaviour, specifically whether they would monitor/observe or call the veterinarian.

Behavioural changes	Number of respondents	Monitor or observe horse	Call a veterinary surgeon	Alternative response	Decision significantly associated with
**Quiet/depressed**	1,543	83% (n = 1,280)	12% (n = 178)	5% (n = 85)	
**Fence/Box walking**	1,544	79% (n = 1,220)	8% (n = 127)	13% (n = 197)	Age of the owner ($p<0.01$) or experience of a colic case in the previous year ($p<0.01$)
**Weight shifting from one foot to another**	1,546	70% (n = 1,075)	16% (n = 241)	24% (n = 230)	
**Pawing at floor (not in response to food or other stimulus)**	1,538	59% (n = 914)	31% (n = 484)	10% (n = 140)	Previous experience of a critical case ($p = 0.025$)
**Rolling >5 minutes or multiple times (4+) in 30 minutes**	1,552	6% (n = 99)	90% (n = 1,390)	4% (n = 63)	If owner had qualifications of college level or higher ($p = 0.048$)
**Restlessly lying down and getting up (4+ times in 30 minute period)**	1,548	9% (n = 134)	88% (1,358)	3% (n = 56)	

**Lying down and getting up (2-3 times in 30 minutes)**	1,546	29% (n = 451)	65% (n = 1,002)	6% (n = 93)	Previous experience of a critical case (p = 0.043)
**Kicking at abdomen (no obvious external stimulus e.g. flies)**	1,546	30% (n = 467)	65% (n = 999)	5% (n = 80)	
**Flank watching**	1,547	45% (n = 689)	50% (n = 770)	5% (n = 88)	
**Inappetant**	1,547	51% (n = 789)	42% (n = 648)	7% (n = 110)	
