# Supplementary material for: Horse owners’ knowledge, and opinions on recognising colic in the horse
Source: Equine Vet J. 2019 Sep 23;52(2):262–7. doi: 10.1111/evj.13173 (PMC7027804; doi:10.1111/evj.13173)
Supplement: Supplementary file 5 — Supplementary item 5: Areas where information is lacking on colic in the horse, identified by horse owners (n = 940) in their horse in an online survey of horse owners’ knowledge and understanding of colic. [file EVJ-52-262-s005.pdf]

BEVA Equine Veterinary Journal

Supplementary Item 5: Use of information and resources by participants in an online survey of horse owners' knowledge and understanding of colic (n = 1,486).

This supplementary item provides further information on horse owners' responses to the question on how they use information and resources.

The majority of participants would ask veterinarians (83%; n = 1,233/1,486) or use the internet (73%; n = 1,151/1,486), followed by books (50%; n = 740/1,486). Other responses were yard owners or trainers (39%; n = 583/1,486), friends and family (31%; n = 455/1,486), paraprofessionals (30%; n = 449/1,486), Pony Club information (6%; n = 84/1,486), phone apps (6%; n = 91/1,486) and posters (4%; n = 53/1,486). Analysis of free text responses (n = 979) detailing specific resources identified 119 different answers. Specific magazines (21 titles), internet (11 websites), books (21 titles) and resources from veterinary practices were most commonly mentioned. Fifteen equine non-veterinary organisations or charities were also named, along with 20 different forums. When asked how often they accessed information on colic, the most frequent response was once every 6 months (32%) (n = 428/1,356). Common answers for 'other' responses (n = 149) included reading about colic when coming across material on it, or if there had been a recent episode.
