# Supplementary material for: Horse owners’ knowledge, and opinions on recognising colic in the horse
Source: Equine Vet J. 2019 Sep 23;52(2):262–7. doi: 10.1111/evj.13173 (PMC7027804; doi:10.1111/evj.13173)
Supplement: Supplementary file 6 — Supplementary item 6: Use of information and resources by participants in an online survey of horse owners’ knowledge and understanding of colic (n = 1486). [file EVJ-52-262-s006.pdf]

BEVA Equine Veterinary Journal

Supplementary Item 6: Areas where information is lacking on colic in the horse, identified by horse owners (n = 940) in their horse in an online survey of horse owners' knowledge and understanding of colic.

This supplementary item provides a list of the areas which were nominated in free text responses by horse owners when asked where they thought information on colic was lacking.

Areas identified by participants where information is thought to be lacking on colic in the horse
**Presentation/recognition of colic**
**Different types/causes of colic**
**What to do whilst a horse is colicing**
**Prevention of colic**
**Normal ranges and gut sounds**
**When to call a vet**
**Treatment of colic**
**Early or subtle symptoms of colic**
**All colic information**
**Information on surgery for colic**
